# Supplementary material for: The causal relationship between the human gut microbiota and pyogenic arthritis: a Mendelian randomization study
Source: Front Cell Infect Microbiol. 2024 Nov 26;14:1452480. doi: 10.3389/fcimb.2024.1452480 (PMC11629706; doi:10.3389/fcimb.2024.1452480)
Supplement: Supplementary file 1 [file DataSheet1.zip › mendelian test/ebi-a-GCST90027530.csv_forest.pdf]

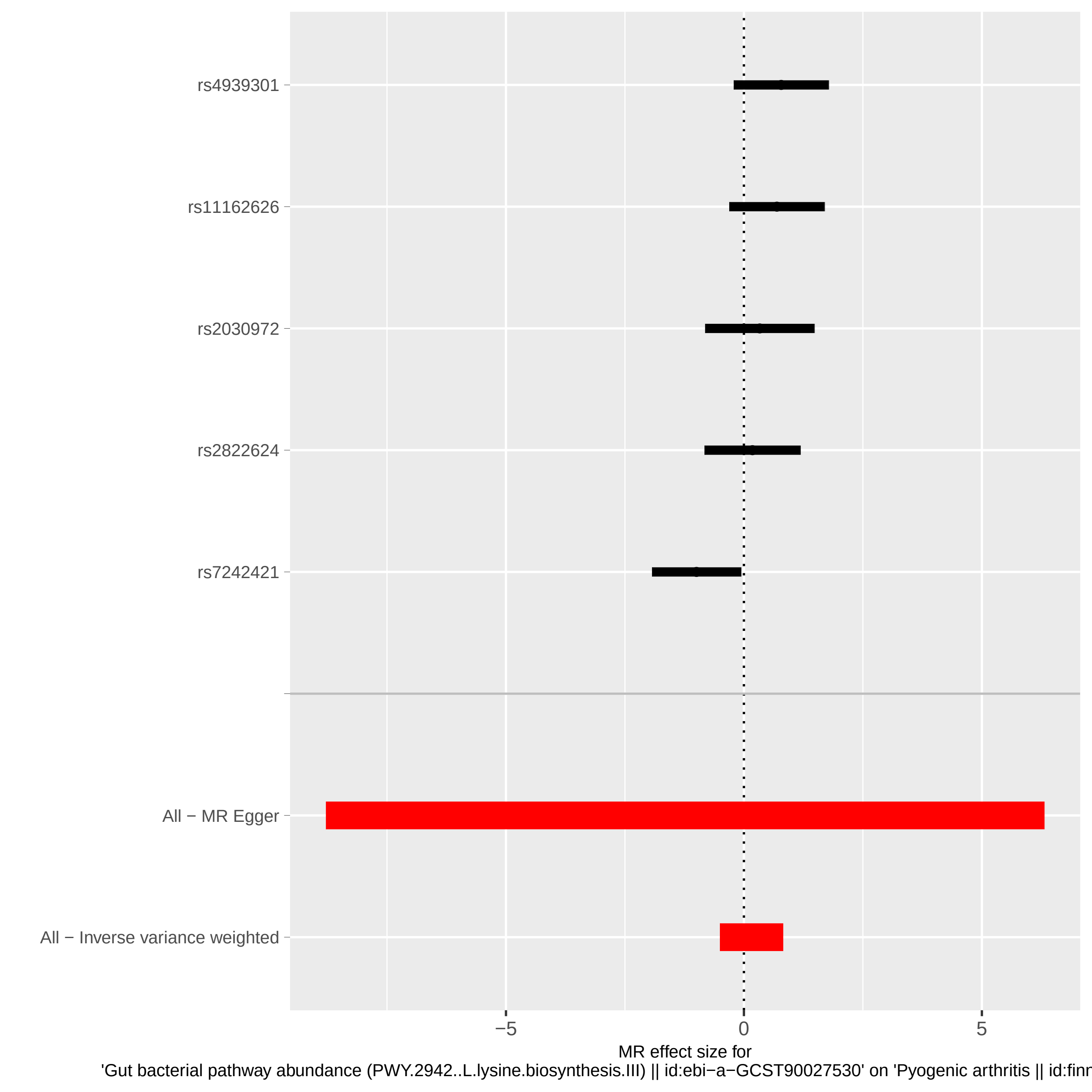
rs4939301
rs11162626
rs2030972
rs2822624
rs7242421
All − MR Egger
All − Inverse variance weighted
−5
0
5
MR effect size for
'Gut bacterial pathway abundance (PWY.2942..L.lysine.biosynthesis.III) || id:ebi−a−GCST90027530' on 'Pyogenic arthritis || id:finn
